# The role of tropical waves in the genesis of Tropical Cyclone Seroja in the Maritime Continent

**DOI:** 10.1038/s41467-023-36498-w

**Published:** 2023-02-15

**Authors:** Beata Latos, Philippe Peyrillé, Thierry Lefort, Dariusz B. Baranowski, Maria K. Flatau, Piotr J. Flatau, Nelly Florida Riama, Donaldi S. Permana, Adam V. Rydbeck, Adrian J. Matthews

**Affiliations:** 1 Institute of Geophysics, Polish Academy of Sciences, Warsaw, Poland; 2 CNRM, Université de Toulouse, Météo-France, CNRS, Toulouse, Languedoc-Roussillon-Midi-Pyrénées France; 3 École Nationale de la Météorologie, Météo-France, Toulouse, Languedoc-Roussillon-Midi-Pyrénées France; 4 Naval Research Laboratory, Monterey, CA USA; 5 Scripps Institution of Oceanography, University of California San Diego, San Diego, CA USA; 6 Agency for Meteorology, Climatology and Geophysics of the Republic of Indonesia (BMKG), Jakarta, Indonesia; 7 Naval Research Laboratory, Stennis Space Center, Mississippi, MS USA; 8 Centre for Ocean and Atmospheric Sciences, School of Environmental Sciences and School of Mathematics, University of East Anglia, Norwich, Norfolk UK

**Keywords:** Natural hazards, Atmospheric dynamics

## Abstract

Tropical cyclone Seroja was one of the first tropical cyclones to significantly impact Indonesian land, and the strongest one in such close proximity to Timor Island. In April 2021 Seroja brought historic flooding to near-equatorial regions of Indonesia and East Timor, as well as impacting Western Australia. Here we show that the unusual near-equatorial cyclogenesis in close proximity to a land mass was due to “perfect storm” conditions associated with multiple wave interactions. Specifically, this was associated with enhanced equatorial convection on the leading edge of a Madden–Julian Oscillation (MJO) event. Within the MJO, the interaction between a convectively coupled equatorial Rossby wave and two convectively coupled Kelvin waves span up the initial vortex and accelerated cyclogenesis. On average, such favorable atmospheric conditions can occur once per year. These results indicate the potential for increased predictability of tropical cyclones over the Maritime Continent.

## Abstract (web summary)

Tropical cyclone Seroja was one of the first cyclones to strike near-equatorial regions of Indonesia and East Timor. The unusual cyclogenesis close to a land mass was due to “perfect storm” conditions associated with multiple wave interactions.

## Introduction

Between March 29 and April 5, 2021, the island of Timor and surrounding islands of Indonesia were hit by torrential rain. These extreme conditions were caused by tropical cyclone (TC) Seroja, which was one of the first TCs to have such a significant impact on Indonesian land^1^. Due to the fact that the low-pressure system remained in the vicinity of Timor for several days, heavy and sustained rainfall caused extensive flooding and landslides on this and neighboring islands. Widespread and devastating damage was reported^2^. It is estimated that at least 272 people were killed by the storm in Indonesia and East Timor, with an economic loss of over $475 million^3^. In addition to causing significant damage in Indonesia and East Timor, Seroja also impacted Western Australia’s Mid West region. The storm reached Category 3 on the Australian Tropical Cyclone Intensity Scale^4^.

Tropical areas, including the Maritime Continent (MC), are subject to heavy rainfalls associated with a number of atmospheric phenomena. Indonesian islands often experience extreme weather related to the passage of equatorial disturbances such as Madden-Julian Oscillation (MJO, e.g.^5,6^) and convectively coupled equatorial waves^7,8^ which trigger natural hazards, including extreme rainfall^9–12^ and floods^13,14^. The area to the north of Australia, over the tropical waters of the MC, is often hit by tropical storms and cyclones^15^; it is home to nearly 15% of global TC activity^16^. However, Indonesia and Timor are rarely influenced by the landfalling TCs. The islands are generally TC-impact-free region^17^. According to the Agency for Meteorology, Climatology, and Geophysics of the Republic of Indonesia (BMKG), within Jakarta Tropical Cyclone Warning Centre area of responsibility Indonesia has experienced ten Tropical Cyclones since 2008. According to the International Best Track Archive for Climate Stewardship (IBTrACS), since 2008, seven low pressure systems (disturbance, tropical low or tropical cyclone) moved in close proximity (200 km) to Timor Island. These were low pressure systems that later developed into tropical storms Errol (2011), Gillian (2014), Magda (2010), Christina (2013), Marcus (2018), Lili (2019), and Seroja (2021). There were no reports of serious damage or injuries associated with these storms except in the case of TC Seroja. Only Seroja met the definition of a tropical cyclone in this region. The proximity to the Equator and the many islands present in the vicinity of the Timor and Savu Seas make cyclogenesis in this region very rare. TCs are usually generated in the open ocean between 10° and 20° equatorial belt^18^. In the Indonesian archipelago, they mostly occur in the Indian Ocean^19^. Therefore, the development of TC Seroja within the Indonesian eastern seas and the serious damage it caused were unusual.

A number of conditions have to be fulfilled in order for a TC to potentially form and develop. Although drivers of formation are basin-dependent, rather than uniform in space and time^20^, there are six known drivers which play a critical role in promoting tropical cyclone formation: warm surface ocean waters, high moisture levels in the low and mid-troposphere, Coriolis force, a weak vertical wind shear, atmospheric instability and large cyclonic relative vorticity in the lower troposphere^21,22^.

The most common location for cyclogenesis is within or close to the Intertropical Convergence Zone (ITCZ)^23^, which serves as a guide for the propagation of equatorial waves, and where pre-existing low pressure and vorticity may exist^24,25^. TC activity is modulated by large scale subseasonal processes, in particular equatorially trapped convectively coupled waves^23,26–33^ and MJO events^34–36^. The TC season over the southern Maritime Continent extends from December to April^16^, overlapping with the highest seasonal activity of MJO^37^. TC genesis locations are usually clustered slightly poleward and a little westward of the center of the MJO convective envelope^15,23,38^. The peak in TC activity near Australia occurs during MJO phases 4–5 and 6–7^39^. Recently, Balaguru et al.^40^ reported that more than 40% of all western Australian 6-hourly TC track locations are found during the period when the active phase of the MJO passes through the MC region (phases 3–5), that is, when the atmospheric conditions such as vertical wind shear, moisture and increased cyclonic vorticity favor cyclogenesis.

At larger scale, a Monsoon Trough, located within the ITCZ, may break down spontaneously and start the cyclogenesis process if it remains unperturbed for a long enough period. Also, an external forcing on the Monsoon Trough, such as tropical waves, can accelerate cyclogenesis^23^.

Several studies have found a link between TC genesis and activity of MJO and equatorially trapped convectively coupled waves in tropical basins across the globe. However, there is an apparent lack of studies which focus specifically on the southern Maritime Continent region. To date, researchers have chosen to explicitly exclude some parts of the MC region^23^ or to split it between the Indian Ocean and the Pacific^41^, which blends the possible local MC environment with that of an open ocean in the Pacific and/or Indian ocean domain. To date, one of the most challenging issues in TC research is tropical cyclogenesis^33,38,42^.

This apparent lack of research emphasis on TC genesis in the southern MC region led us to embark on a case study of TC Seroja. The main goal is to understand the multi-scale atmospheric conditions leading to the genesis and subsequent development of TC Seroja. In particular, we would like to understand the role of convectively coupled tropical waves and MJO. Section 2 presents detailed results of the TC Seroja genesis. Section 3 presents discussion and conclusions. The data and methodology used are described in Section 4.

## Results

### Meteorological history

**Fig. 1:**
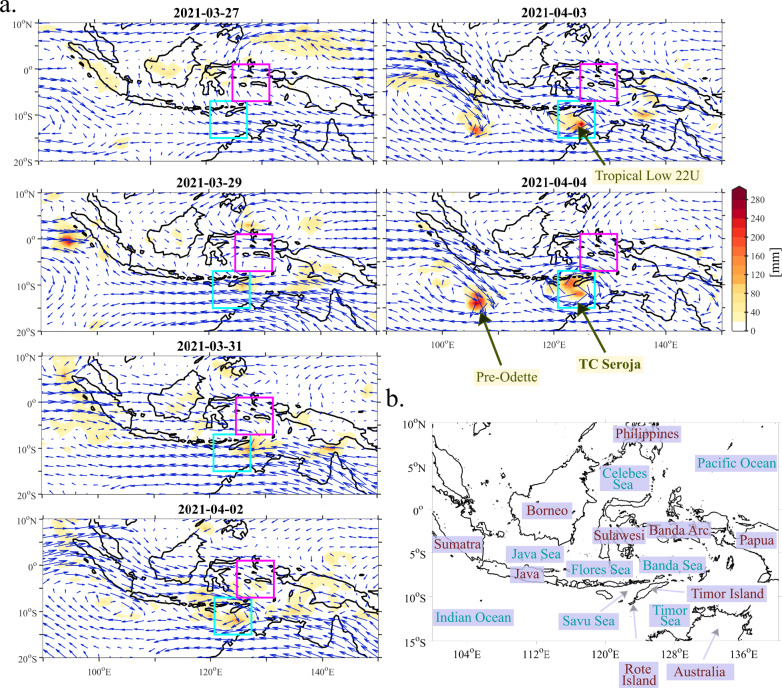
Meteorological conditions during the development of Tropical Cyclone Seroja over the Maritime Continent. **a** Daily mean 850 hPa ERA-5 winds (arrows) and daily accumulated GPM precipitation based on combined microwave-IR retrievals (shaded in mm) for selected days in March and April 2021. The longest vector corresponds to a wind speed of 28 m s^−1^. The two boxes which are analysed in the paper are marked in pink (pre-Seroja box) and blue (Seroja box), **b** the western and central Maritime Continent region.

**Fig. 2:**
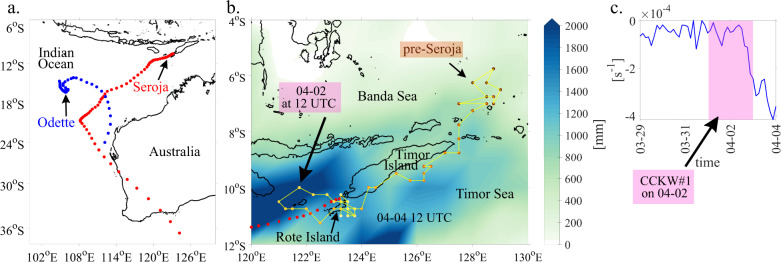
Seroja and pre-Seroja tracks. **a** Tropical Cyclone (TC) Seroja (red dots) and TC Odette (blue dots) tracks obtained from the International Best Track Archive for Climate Stewardship (IBTrACS). Data every 3 h starting on April 4, 12 UTC, **b** backward trajectory (precursor) of TC Seroja based on ERA-5 relative vorticity (dotted line, data every 3 h starting on March 28, 0 UTC) and accumulation of precipitation anomalies (GPM data) between March 28 and April 7 (shaded), **c** ERA-5 relative vorticity of TC Seroja precursor (before it is named) integrated between 950 hPa and 500 hPa; data points every 3 h. Pink box indicates when the first Convectively Coupled Kelvin Wave occurred.

In early April 2021, a slow-moving low pressure system was developing near the southwestern end of the island of Timor (Fig. 1). On April 3 2021, the Australian Bureau of Meteorology (BoM) recognised it as a Tropical Low 22U. The low intensified into Category 1 Tropical Cyclone by April 4 and was given the name Seroja—meaning lotus flower—by the Tropical Cyclone Warning Centre (TCWC) Jakarta, while it was passing about 95 km north-northwest of Rote Island at 10.4°S, 123.2°E^43^. The storm moved away from Indonesia and East Timor and underwent an interaction with Tropical Cyclone Odette, present in Seroja’s vicinity (Fig. 2a). The interaction, known as the Fujiwhara Effect^44^, caused their centers to begin orbiting around a point between the two systems. TC Seroja was dominating Odette, absorbing it into its circulation on April 10. The interaction complicated forecasting the paths of the cyclones, as it was not clear beforehand which TC would persist. Some of the aspects of the binary interaction that were shown in^45^ were present in this case (Approach, Capture and Merge).

On April 11, Seroja made landfall on the western coastline of Western Australia as a Category 3 severe tropical cyclone. Later that day, Seroja began weakening and accelerating southeastward. On April 12, Seroja emerged off the southern coast of Western Australia while undergoing an extratropical transition (Fig. 2a).

### Large-scale environmental conditions

At the time of Seroja’s development, the environmental conditions were favorable for cyclogenesis with weak vertical wind shear and robust poleward outflow at 200 hPa into the westerlies (not shown). The sea surface temperatures (SSTs) were around 29–30 °C, which is well above the common formation threshold of 26.5 °C^21^ and above the SSTs of 27.5–28.5 °C which are typically observed during the genesis of the majority of Southern Hemisphere storms^20^. The high SSTs were consistent with the La Niña conditions favorable for cyclogenesis in the vicinity of Timor^39^.

Trade winds were anomalously strong so that westerly bursts connected with equatorial waves (Section 2.4) produced strong horizontal shear and vorticity. This happened within the so-called Near Equatorial Trough that occurs during the transition season between monsoons (Fig. 1a). At that time, the cross-equatorial flow which is typical for a Monsoon Trough was not observed^46,47^.

### Intraseasonal variability

The Madden-Julian Oscillation propagation through the Maritime Continent was observed during late March and early April 2021. The RMM index^48^ indicates an active MJO starting in mid-March during phase 1, while the OLR MJO index (OMI)^49^ (not shown) MJO amplitudes larger than 1 only after March 29 during phase 3. On April 4, when Seroja became a Tropical Cyclone, MJO was in phase 4 (RMM index) or 5 (according to OMI) indicating that the active phase of the MJO was in the MC region. The precursor of Seroja (see Section 2.5) developed on the leading edge of the MJO, ahead of the convective phase.

**Fig. 3:**
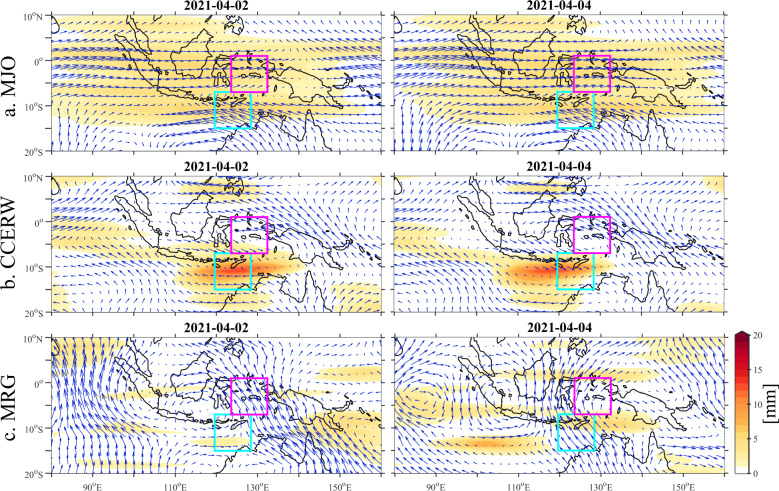
Time evolution of GPM precipitation anomalies (shaded) and ERA-5 850 hPa wind anomalies (arrows) during the TC Seroja genesis. Wavenumber-frequency filtered anomalies for **a** Madden-Julian Oscillation, **b** Convectively Coupled Equatorial Rossby Waves, and **c** Mixed Rossby-Gravity Waves. All GPM anomalies are in mm and only positive anomalies are plotted. The longest vector corresponds to a wind speed of 5.5 m s^−1^.

**Fig. 4:**
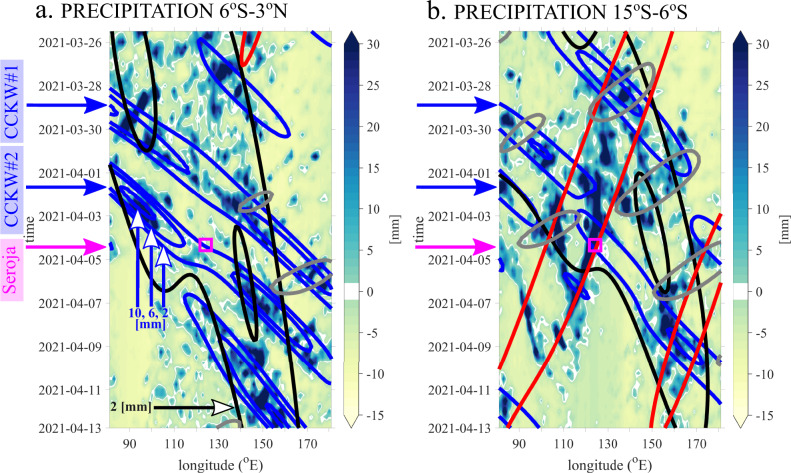
Time-longitude Hovmöller diagrams based on GPM precipitation data. **a** 6 hourly accumulated GPM precipitation anomalies [mm] averaged between 6°S and 3°N (color shading). Line contours show amplitudes of selected equatorial modes that have been wavenumber-frequency filtered: Convectively Coupled Kelvin Wave (blue lines), Convectively Coupled Equatorial Rossby Waves (red lines), Madden-Julian Oscillation (black lines), Mixed Rossby-Gravity Waves (grey lines). Data averaged over the same domain as anomalies, contours from 2 mm, every 4 mm, **b** same as **a** but for different domain; data averaged between 15°S and 6°S. Pink squares at the level of pink arrows indicate timing and location of pre-Seroja vortex developing into Tropical Cyclone (April 4, 12 UTC at 123.2°E).

Figure 3a shows GPM precipitation and ERA-5 850 hPa winds filtered for the MJO. The eastward propagation of an established large-scale MJO envelope can be observed with positive precipitation anomalies ahead of a marked westerly anomaly at the equator. This is further confirmed on Hovmöller diagrams (Fig. 4), where MJO (black contours) is observed in precipitation.

### The role of tropical waves in Seroja’s cyclogenesis

In addition to the MJO (black contours in Fig. 4), the Seroja genesis region was under the influence of equatorial waves: two eastward-propagating convectively coupled Kelvin waves (CCKWs) (blue contours), and a westward-propagating convectively coupled equatorial Rossby wave (CCERW) (red contours). Mixed Rossby-Gravity wave (MRG) is also seen (grey contours at Fig. 4).

**Fig. 5:**
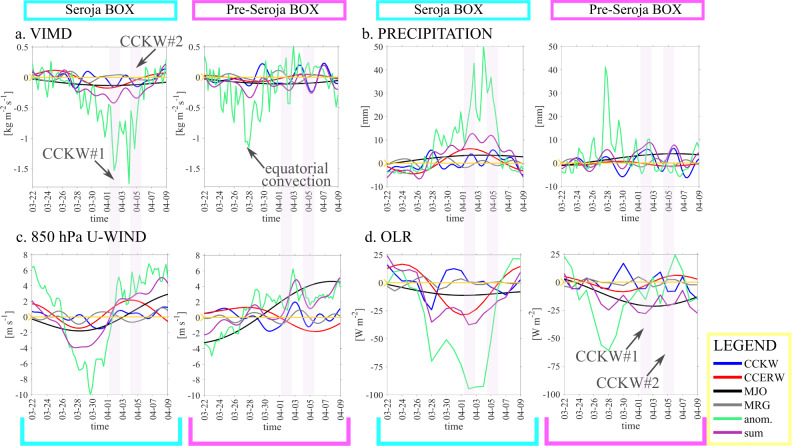
Time series at two locations: Pre-Seroja (125–132°E, 6°S–3°N) box and Seroja (120–128°E, 15–6°S) box for the period of March 22 to April 10, 2021. **a** 6-hourly ERA-5 vertically integrated moisture divergence (VIMD), **b** 6-hourly GPM precipitation, **c** zonal component of 850 hPa wind (U-WIND), and **d** outgoing longwave radiation (OLR). Color lines indicate Convectively Coupled Kelvin Waves (CCKW, blue), Convectively Coupled Equatorial Rossby Waves (CCERW, red), Madden-Julian Oscillation (MJO, black), Mixed Rossby-Gravity Waves (MRG, grey), green lines depict anomalies and the sum of all waves is presented in purple. Two pink boxes indicate periods of two sequential CCKW occurrence.

In agreement with the approaching convective phase of MJO, total zonal wind anomalies at 850 hPa shift from easterlies to westerlies (Fig. 5c): first at the equator (pre-Seroja box) and later in the south (Seroja box). In the preSeroja box, the total zonal wind speed anomaly slowly but regularly increases (Fig. 5c). The anomaly reaches its maximum when all waves, apart from CCERW, are in phase (2–3 of April).

**Fig. 6:**
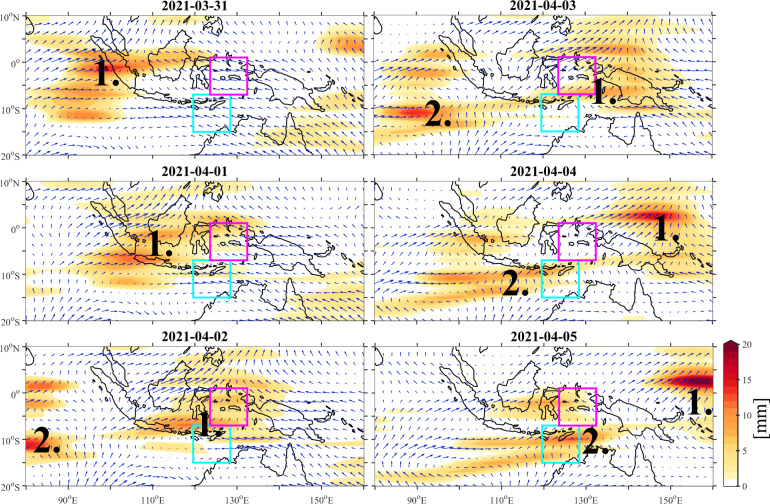
Time evolution of GPM precipitation anomalies (shaded) and ERA-5 850 hPa winds (arrows) during the Tropical Cyclone Seroja genesis. Anomalies are wavenumber-frequency filtered for Convectively Coupled Kelvin Waves. All GPM anomalies are in mm and only positive anomalies are plotted. The longest vector corresponds to a wind speed of 5.5 m s^−1^.

Further details of the influence of CCKWs on Seroja’s development are shown in Fig. 6. There were two Kelvin waves that propagated over the Maritime Continent and contributed to the TC genesis. Their timing coincides with devastating floods reported at that time in East Timor^50^. CCKW#1 had maximum precipitation over the Indian Ocean on March 30 and arrived over Sumatra on March 31. Then, on April 1 and 2, CCKW#1 deflected slightly south off the Equator after being partially blocked by the mountain range on Sumatra (Fig. 6). This deflection was crucial for structuring the convection over Timor by positioning the CCKW westerly wind anomaly in the Java sea. Recently, Matthews^51^ showed that the meridional gradient of the zonal wind anomaly results in significant relative vorticity south of a CCKW axis. This study shows that over the Indian Ocean, a major contribution to the vorticity tendency comes from CCKW divergence acting on background environmental vorticity. A similar mechanism can be observed during development of TC Seroja when CCKW#1 interacts with vorticity embedded within CCERW. Integrated relative vorticity between 950 hPa and 500 hPa tracked in the pre-Seroja vortex (Fig. 2c) shows sudden deepening of the vortex during interaction with CCKW#1 westerlies. The timing of CCKW#1 occurrence over the area is estimated based on precipitation and 850 hPa winds (Fig. 6) and indicated by the pink box on Fig. 2c. CCKW#1 westerlies estimated at 2–3 m s^−1^ strengthen already existing pre-Seroja vortex and spin up its rotation, but also helped the environmental vorticity to propagate to the south, over the Seroja box. Animation of integrated relative vorticity and Himawari-8 infrared data (band 13) can be found by following the link: 10.6084/m9.figshare.20800054.v1^52^.

**Fig. 7:**
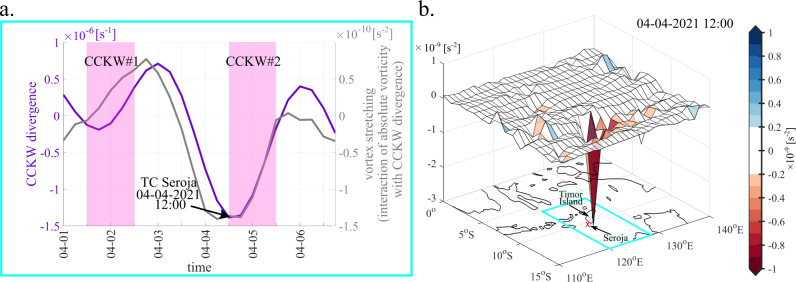
Convectively Coupled Kelvin Wave (CCKW) influence on vorticity. **a** CCKW low-level (850 hPa) divergence (left axis) together with vortex stretching: low-level CCKW divergence acting on absolute vorticity (right axis), averaged over Seroja box. Two pink boxes indicate periods of two sequential CCKW occurrences. **b** Vortex stretching (colour-shaded): low-level CCKW divergence acting on absolute vorticity at 12 UTC April 4, 2021. Red sign on the underlying map shows the location of Tropical Cyclone Seroja vortex. The box which is analysed on **a** is marked in **b**.

In terms of Kelvin wave-driven convergence (negative divergence) acting on absolute vorticity^51^, both CCKW were important, but CCKW#2 was even more impactful than CCKW#1. Figure 7a, b shows the vortex stretching term—the interaction of absolute vorticity with low-level anomalous divergence associated with CCKWs. The timing of Kelvin waves activity over the area are estimated based on precipitation and 850 hPa winds, independently, and indicated by the pink boxes. It is worth noting, that the timing of the pre-Seroja vortex developing into TC Seroja corresponds to the maximum of the CCKW-driven convergence and the greatest impact of the CCKW#2 on vorticity (Fig. 7a). On April 4, 2021, TC Seroja was located in the area of the maximum CCKW-driven vorticity tendency supportive of cyclonic rotation (Fig. 7b). This shows that CCKW#2 was an external forcing that accelerated cyclogenesis.

Figure 5a shows vertically integrated moisture divergence (VIMD) filtered for tropical waves. We can see that vertically integrated moisture convergence was one of the main features of each of the two propagating waves and contributed to intensification of convection. On April 2 and 5 CCKW-driven moisture convergence accounts for about 40–45% of the sum of all waves. Figure 5b shows precipitation filtered for tropical waves. The sum of all waves (purple line) and CCKW (blue line) are predominantly in phase with the anomalous total (green line) in the Seroja and pre-Seroja boxes indicating the role of these disturbances in creating the conditions favorable for heavy precipitation.

Convectively coupled equatorial Rossby wave also significantly contributed to the general circulation and precipitation pattern in the Seroja region. Figure 3b shows the westward propagation of CCERW-filtered GPM precipitation and ERA-5 850 hPa winds. Arriving from the Pacific Ocean, CCERW was strengthened over the MC. This strong CCERW signal was also visible in unfiltered data, i.e. at low-level winds (Fig. 1). It is clearly seen in precipitation (Fig. 4). CCERW contributed to the enhancement of the equatorial convection that initiated Seroja cyclogenesis on March 28 creating vortex that subsequently propagated southwest over Timor island. The interaction between both Kelvin Waves and this strong CCERW was observed (Fig. 4) as well. Coincidence of such interactions^11,14,53^ are on occasion supportive for the extreme weather events.

Mixed Rossby-Gravity wave contributions were moderate in precipitation (Figs. 3c and 4) but significant for the wind pattern (Fig. 3c) and water vapour flux (not shown). On April 2, MRG cyclonic flow was supportive of intensification of TC Seroja. However, on April 4 MRG was destructive for a tropical low intensification (Fig. 3c). As indicated by vertically integrated water vapour flux (VIWVF), on April 2–5, MRG played an important role in moisture advection. The sum of all waves’ northward component of VIWVF is predominantly in phase with the anomaly in the Seroja box with MRG dominating the anomalous total (not shown).

**Fig. 8:**
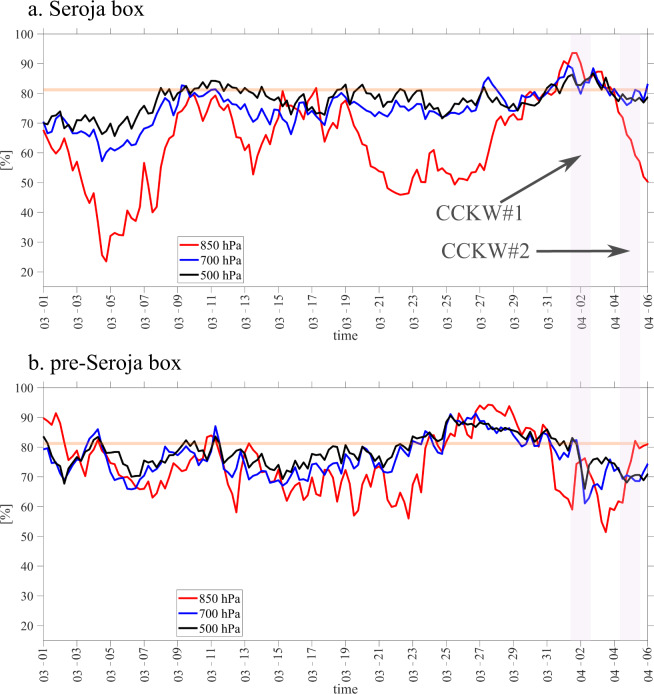
Relative humidity changes before and after Tropical Cyclone Seroja. 6-hourly ERA-5 relative humidity at three levels in % averaged over **a** the Seroja box between 120–128°E, 15–6°S and **b** the pre-Seroja box between 125–132°E and 6°S–3°N. Horizontal orange line indicates level of 81% relative humidity. Two pink boxes indicate periods of two sequential Convectively Coupled Kelvin Wave occurrence.

The high moisture levels which are required for successful storm genesis can be enhanced by tropical waves. For Southern Hemisphere storms, the mean relative humidity (RH) observed during the TC genesis is 81% at 850 hPa^20^, which is in agreement with our case study. At the TC genesis box (Fig. 8a), the highest values of low-level RH correspond to the passage of CCKW#1 (April 1 and 2). On April 4 at 0 UTC relative humidity reached 82% at 700 hPa, 80% at 850 hPa and 79% at 500 hPa (Fig. 8a). At that time, low (850 hPa) and mid-levels (700, 500 hPa) were very humid; in the several days preceding April 4, RH at all these levels were above 80% and there was an overall slight upward trend in mid-levels. The middle troposphere was moistened by the widespread moderately deep convection associated with approaching MJO^54^. The signature of CCERW is also clear based on Fig. 5a, d with a dry phase from March 22 to 28 and a moistening phase from March 28 to April 4. After the TC moved away, there was noticeable drying at 850 hPa (Fig. 8a).

In the pre-Seroja box (Fig. 8b), the overall maximum of RH at all levels was observed on March 26–29 when the equatorial convection developed. This moistening partly resulted from the vertically integrated moisture convergence observed at that time (negative divergence in Fig. 5a).

### Cyclogenesis

Development of Seroja can be traced to the local convective system that developed on the Equator around March 28 in the area of high SST ahead of the approaching MJO (see Section 2.4). Positive SST anomalies in this area were likely induced by MJO suppressed regime and increased insolation related to clear skies and low surface winds preceding propagating MJO^40,55^. From mid-March to 23 of March, OLR anomalies in the pre-Seroja area were positive, indicating suppressed convection and rainfall. Later, on March 29, after the breakdown of the equatorial convection^56^, the southern vortex propagated southwest and merged with the equatorial Rossby wave which was propagating over this region. The precipitation and OLR patterns in Fig. 5b, d indicate a convective peak associated with this local disturbance. In the pre-Seroja domain, the 200 hPa peak in the horizontal divergence can also be observed (not shown).

The diurnal cycle of convection over Papua ahead of the MJO convective envelope contributed to the development of the equatorial convective burst observed on March 28. The animation (10.6084/m9.figshare.20800054.v1^52^) of integrated vorticity and Himawari-8 infrared channel shows the enhancement of the diurnal cycle that occurred before March 28 along the upwind slope of the mountains in New Guinea. This conforms with^57^, who demonstrated that precipitation is enhanced over the islands of the MC prior to the arrival of the MJO.

## Discussion

In this study we have examined meteorological drivers that led to genesis of the Tropical Cyclone Seroja. Developing in April 2021 over the Maritime Continent, it brought historic flooding and landslides to southern Indonesia, East Timor and also impacted Western Australia’s Mid West region. Seroja was one of the first TCs to have a significant impact on Indonesian land and the strongest one in such close proximity to Timor Island.

**Fig. 9:**
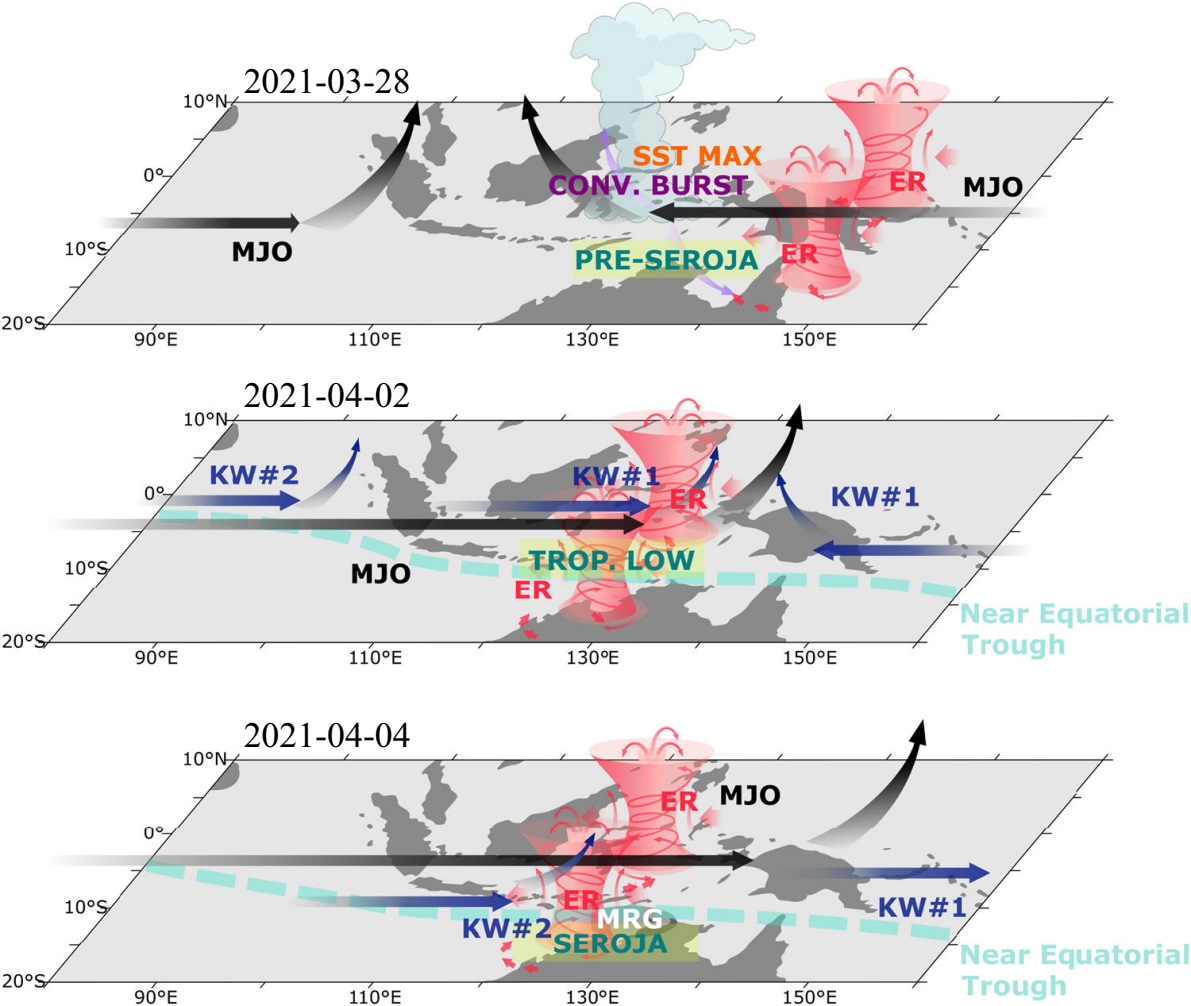
A conceptual diagram of meteorological drivers of TC Seroja. Abbreviations are as follows: SST MAX—maximum of the Sea Surface Temperatures; CONV. BURST—Convective Burst; KW—Convectively Coupled Kelvin Wave; ER—Convectively Coupled Equatorial Rossby Wave; MJO—Madden-Julian Oscillation; TROP. LOW—Tropical Low; MRG—Mixed Rossby-Gravity Wave.

A conceptual diagram of meteorological conditions leading to the genesis of TC Seroja is presented in Fig. 9. The interactions between different spatial and temporal-scale drivers of convection are shown. The genesis region of the Timor and Savu Seas is depicted and the combination of ocean forcing ahead of MJO and the interaction between equatorially trapped modes is presented. TC Seroja genesis started with deep equatorial convection on March 28, 2021 which was preceded by warm sea surface anomalies in that region and an increased diurnal cycle over Papua. This deep equatorial convection developed in the area of large insolation related to clear skies and low surface winds on the leading edge of MJO ahead of its convective phase^55^. This equatorial convection triggered a westward-propagating disturbance associated with the Gill–Matsuno mechanism^56^ and generated anomalous cyclonic circulation, leading to the convergence of moisture and increased precipitation^58^. In the case of TC Seroja, interaction between the convectively coupled equatorial Rossby wave and two convectively coupled Kelvin waves which were embedded within the larger-scale envelope of the MJO was crucial and provided a supportive environment for its genesis. The initial deep equatorial convection moved southwest and was boosted by the environmental cyclonic vorticity associated with convectively coupled equatorial Rossby wave. Each of the two Kelvin Waves that arrived over the Maritime Continent had a unique contribution to this event, in structuring the convection, winds and precipitation patterns. In particular, during CCKW#1 westerly burst occurrence a sudden deepening of the pre-Seroja vortex was observed.

The development and consequences of TC Seroja were unusual for this part of the world (for detailed information, see Supplementary text and Supplementary Figs. 1–3). TC Seroja was triggered by a perfect storm of equatorial waves and made landfall over Indonesian land, relatively close to the Equator.

Calculations show that on average, co-occurrence of MJO, CCKW, ER and the relevant “perfect storm conditions” can occur once per year. We used GPM precipitation data to choose days of wave-filtered precipitation exceeding 1 standard deviation threshold. In the pre-Seroja box, there were 35 days of cooccurrence of CCKW, MJO and ER, while in the Seroja box there were 51 days of such co-occurrence. Days of the waves’ activity have been further clustered into individual events. Using such identification criteria, we estimated that during 13 years in the pre-Seroja box there were 13 events of co-occurrence of MJO, CCKW and ER and 15 in the Seroja box. In the case of Seroja, CCKW exceeded 2 std and ER exceeded 3 std. This shows that the development of Seroja within the waves was unique for this area. However, it’s difficult to state whether the sole existence of the waves is crucial or whether a specific amplitude of their anomaly has to be reached, as there are no similar cases of TCs to look at.

The question arises: to what extent could such strong storms in Indonesia become more common due to the climate change? This is a topic of current debate^59–61^. Climate change could drive some of the physical precursors discussed in this paper. For example, enhanced activity of the equatorial convectively coupled waves, especially the convectively coupled Kelvin waves, may contribute to increased cyclogenesis near the Equator as discussed here. Using OLR and brightness temperature observations, it was shown^62^ for the period from 1980–2016 that Madden–Julian Oscillations and equatorial Rossby waves were losing a frequency–wavenumber power while Kelvin waves, mixed Rossby–gravity waves, and tropical disturbance–like wave activity were gaining power. Recent study^63^ based on the OLR data for the 40 year period from 1979 to 2018 indicates an increasing trend of equatorial waves in the boreal winter season over the Indian Ocean and West Pacific and large heterogeneity of trends in space and time. Future predictions are a concern. On the other hand, climate predictions project that the MJO amplitude will increase^64–67^ and that the amplitudes of Kelvin waves, mixed Rossby–gravity waves, and westward-propagating inertia-gravity waves^68^ may increase as well. How these predictions translate into the number, trajectories and intensities of TCs remains unknown. Thus, looking at tropical waves in the warmer climates may provide some insight into this complex problem.

## Methods

### Satellite data

Half-hourly Integrated Multisatellite Retrievals for Global Precipitation Mission (IMERG) Final Precipitation L3 V06 products^69^ were used. The data were on a 0.1° × 0.1° latitude-longitude grid for the 1 January 2009–31 December 2021 period. Gridded daily outgoing longwave radiation (OLR)^70^ on a regular 2.5° × 2.5° latitude-longitude grid for the same period as IMERG data was used as a proxy for convection. Additionally, Himawari-8 10-min infrared brightness temperature data at regular grid (L1 level) were utilized at their native spatial resolution of 2 km, for 24 March 2021–10 April 2021. Spectral band 13, representing brightness temperature at central wavelength of 10.45 µm with 0.30 µm was used. These data represent temperature of cloud tops.

### Reanalysis data

European Centre for Medium Range Weather Forecasts (ECMWF) Fifth Generation Reanalysis (ERA-5) reanalysis data (zonal and meridional wind, vertically integrated moisture divergence, divergence, relative vorticity, relative humidity) for the 1 January 2009–31 December 2021 period were obtained on a regular 0.25° × 0.25° latitude-longitude grid at several pressure levels with hourly temporal resolution.

### Calculation of daily anomalies and equatorial wave filtering

Daily and 6-hourly anomalies of all relevant meteorological data were computed by removing the seasonal cycle, defined as the mean plus the first three annual harmonics fitted to an average annual cycle calculated for the 2009–2021 period. Meteorological estimates were further filtered in zonal wavenumber and frequency domain to isolate specific modes of variability: a wavenumber–frequency spectral analysis^7^ was performed for all latitudes in the meridional band of 20°S–20°N for daily and 6-hourly anomalies. The dispersion curves relevant to each type of equatorial wave were used. Convectively coupled Kelvin waves (CCKWs) were defined using equivalent depths of 8–90 m, periods of 2.5–17 days and eastward propagation at zonal wavenumbers 1–14^71^. Mixed Rossby–Gravity (MRG) waves were defined as westward– propagating disturbances with zonal wavenumbers 1–10, periods 3–96 and equivalent depths of 8–90 m^7^, while convectively coupled equatorial Rossby wave (CCERW) filter includes westward-propagating zonal wavenumbers 1–10, periods 9.7–48 days and equivalent depths of 5–9 m^25^. The wavenumber-frequency filtering for the MJO mode retained eastward-propagating signals with zonal wavenumbers 1–9 and periods of 30–96 days^8^. The state of intraseasonal variability was assessed by Realtime Multivariate MJO index (RMM)^48^, together with the OLR MJO index (OMI)^49^.

Tropical cyclones can contribute to the total variance at the shorter wavelengths of the westward-moving waves. Therefore, the TC-removing algorithm by Schreck et al.^72^ was tested. In the case of Seroja, removing the TC-related signal as in Schreck et al.^72^ made little impact on the propagation and structure characteristics of the tropical waves, so the method was not applied.

### Tropical cyclone track and its precursor

Tropical cyclones that occur within the Southern Hemisphere to the east of 90°E are officially monitored by one or more tropical cyclone warning centres: the Fiji Meteorological Service, New Zealand’s MetService, Papua New Guinea’s National Weather Service, the Agency for Meteorology, Climatology, and Geophysics of the Republic of Indonesia (BMKG) and the Australian Bureau of Meteorology (BoM). Within the region a tropical cyclone is defined as being a non-frontal low-pressure system of synoptic scale that develops over warm waters, with a definite organized wind circulation and 10-min sustained wind speeds of 34 kn (63 km/h; 39 mph) or greater near the centre^73^. Australian tropical cyclone intensity scale is used, which measures tropical cyclones using a five category system based on 10-min maximum sustained winds^73^.

TC Seroja best track data were obtained from the International Best Track Archive for Climate Stewardship (IBTrACS)^74^. However, IBTrACS defines location of a TC once it reached sufficient threshold. In the case of TC Seroja, it was April 4 at 12UTC when its center was located off the south-west tip of Timor island. In order to identify and subsequently follow precursor of TC Seroja, a backward trajectory, based on ERA-5 relative vorticity at individual pressure levels and integrated between 950 hPa and 500 hPa, has been constructed. The backward trajectory was initiated at 12UTC on April 4, at the first location provided by the IBTrACS. Then, the local maximum relative vorticity is detected every hour backward in time within a 1° × 1° box around the previous point. These new coordinates replace the initial position and the procedure is repeated until maximum vorticity is no longer detectable as a persisting feature for more than 6 h. As a result, a track of TC Seroja precursor is identified.

## Supplementary information

Supplementary Information

## Data Availability

Tropical cyclone tracks were obtained from NOAA’s International Best Track Archive for Climate Stewardship (IBTrACS) through their web site at ncdc.noaa.gov/ibtracs. The IMERG precipitation data were supplied by the National Aeronautics and Space Administration through their web site gpm.nasa.gov. Research product of the Himawari L1 Gridded Data (produced from Himawari-8) that was used in this paper was supplied by the P-Tree System, Japan Aerospace Exploration Agency (JAXA). This dataset is available at ftp.ptree.jaxa.jp/jma/netcdf, after registration at http://www.eorc.jaxa.jp/ptree/index.html. The ERA-5: the fifth generation of ECMWF atmospheric reanalyses of the global climate was obtained from Copernicus Climate Change Service Climate Data Store through their web site at cds.climate.copernicus.eu. The univariate OLR-based MJO time series indices (OMI) were supplied by the National Oceanic and Atmospheric Administration (NOAA) through their web site at psl.noaa.gov/mjo/mjoindex/omi.1x.txt. The real-time multivariate MJO (RMM) time series indices were supplied by the Australian Bureau of Meteorology through their web site at poama.bom.gov.au/project/maproom/RMM/.

## Code availability

The MATLAB and Python codes used for processing papers’ data are available through the corresponding author, i. e. Beata Latos (blatos@igf.edu.pl).
