# Supplementary material for: The role of tropical waves in the genesis of Tropical Cyclone Seroja in the Maritime Continent
Source: Nat Commun. 2023 Feb 15;14:856. doi: 10.1038/s41467-023-36498-w (PMC9932074; doi:10.1038/s41467-023-36498-w)
Supplement: Supplementary file 1 — Supplementary Information [file 41467_2023_36498_MOESM1_ESM.pdf]

Supplementary information for
The role of tropical waves in the genesis of Tropical Cyclone Seroja in the Maritime Continent

Beata Latos[1]*, Philippe Peyrillé[2], Thierry Lefort[3], Dariusz B. Baranowski[1], Maria K. Flatau[4], Piotr J. Flatau[5], Nelly Florida Riama[6], Donaldi S. Permana[6], Adam V. Rydbeck[7] and Adrian J. Matthews[8]

[1*]Department of Atmospheric Physics, Institute of Geophysics Polish Academy of Sciences, 64 Ksiecia Janusza Street, Warsaw, 01-452, mazowieckie, Poland.
[2]CNRM, Université de Toulouse, Météo-France, CNRS, 42 Avenue Gaspard Coriolis, Toulouse, 31057, Languedoc-Roussillon-Midi-Pyrénées, France.
[3]École Nationale de la Météorologie, Météo-France, 42 Avenue Gaspard Coriolis, Toulouse, 31057, Languedoc-Roussillon-Midi-Pyrénées, France.
[4]Naval Research Laboratory, 7 Grace Hopper Ave, Monterey, 93943, CA, USA.
[5]Scripps Institution of Oceanography, University of California San Diego, 8622 Kennel Way, La Jolla, San Diego, 92037, CA, USA.
[6]Agency for Meteorology, Climatology and Geophysics of the Republic of Indonesia, Jl. Angkasa I, No.2 Kemayoran, Jakarta, 10610, Jakarta, Indonesia.
[7]Naval Research Laboratory, Stennis Space Center, 1005 Balch Boulevard, John C. Stennis Space Center, 39529, MS, USA.
[8]Centre for Ocean and Atmospheric Sciences, School of Environmental Sciences and School of Mathematics, University of East Anglia, Norwich Research Park, Norwich, NR4 7TJ, Norfolk, UK.

*Corresponding author. E-mail: blatos@igf.edu.pl

Supplementary text

In a broad region where TC Seroja developed (110 – 140˚E, 0 – 12˚S), 43 TCs were initiated between 2000 and 2021, according to IBTrACS data. Individual tracks show that most of those storms originated between 130˚E and 140˚E, and propagated south-westward before recurving westward towards the open Indian Ocean, or south-eastward towards the north-west coast of Australia. There are also TCs that move eastward toward Gulf of Carpentaria and can affect north Australia and New Guinea. TC Seroja is an outlier (Supplementary Fig. 1). It not only developed further west than most TCs in the region, it also made landfall in Australia as the south-most TC after year 2000. TCs tend to move over the Timor Sea, about 1/3 of a distance between Australia and Indonesia, and primarily affect north coast of Australia, near Darwin/Tiwi Islands around 131˚E. The second area of relatively frequent landfalls is observed further east on northern coast of Australia, between 123˚E and 128˚E. Islands within the Maritime Continent are affected sporadically (Fig. 2). Analysis of TC initiation location according to the first data entry for each TC in IBTrACS data (Fig. 3) shows that TCs tend to develop between latitudes 9˚S and 11˚S. Zonal distribution shows double maxima at 129˚E and 138˚E. TC Seroja is an outlier due to longitude of its initiation (123˚E), but not its latitude (10˚S). It should be noted that this analysis is based on a first data-point of each TC track and does not account for precursors. In case of TC Seroja the precursor originated at 128˚E, 6˚S, which matches the one of peaks in distribution of initiation longitude, but it is on the edge of typical initiation latitude. As result, TC Seroja can be seen as an unusual TC, for which reliable forecasting of development and track was challenging.

Supplementary Figures

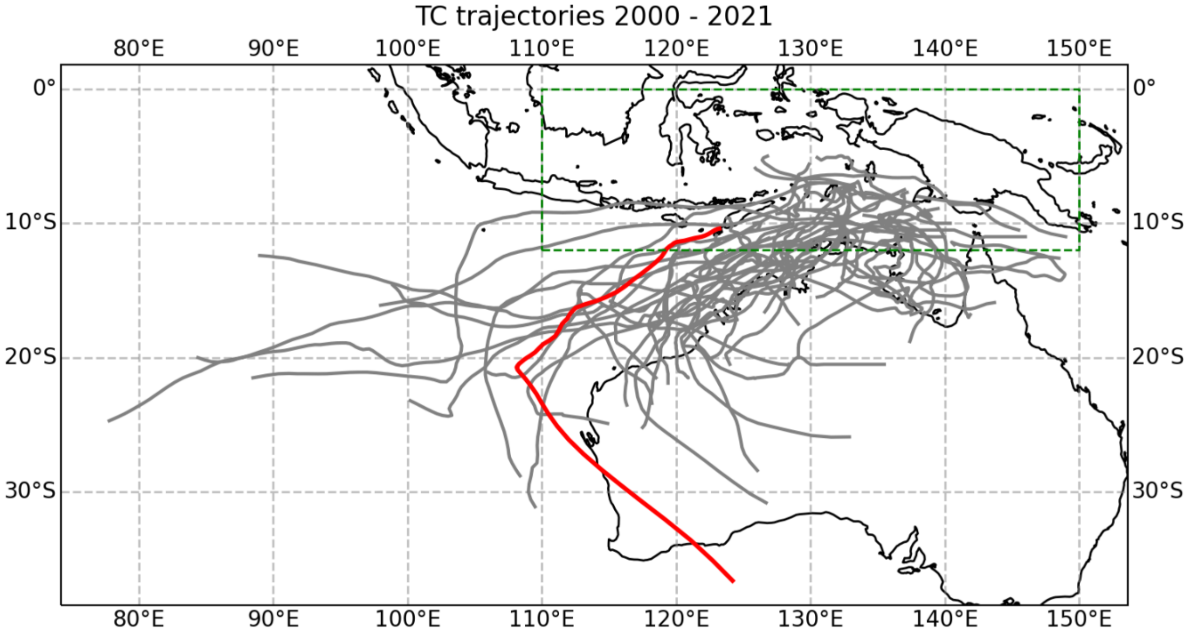

Supplementary Figure 1 Trajectories of TCs that originated within the region defined by a box bounded by 110 ˚E, 140˚E, equator and 12˚S (green dashed line) between 2000 and 2021. Trajectory of TC Seroja marked with the red line. TC tracks obtained from the International Best Track Archive for Climate Stewardship (IBTrACS).

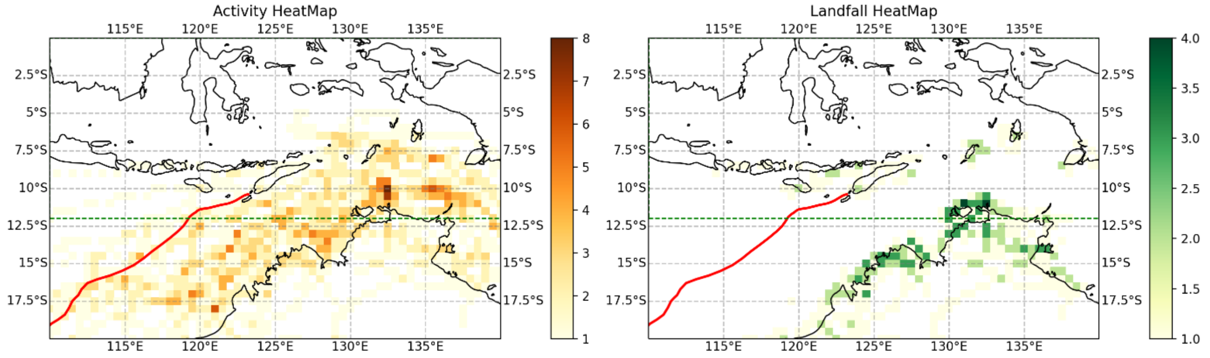

Supplementary Figure 2 Heatmaps of TC activity (left) and TC landfalls between 2000 and 2021 based on IBTrACS data. Landfall is based on minimum distance from a coast, which had to be less than 50km to be counted.

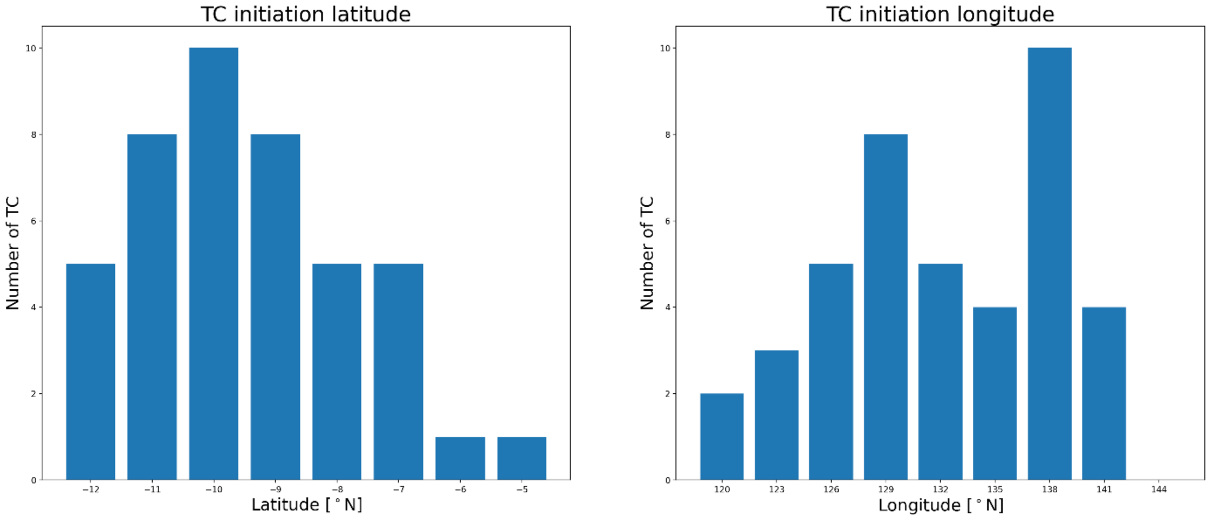

Supplementary Figure 3 Histograms of TC initiation latitude (left) and longitude (right) for all TCs that developed inside the box bounded by 110°E, 140°E, equator and 12°S between 2000 and 2021.
